# Prevalence of human T-cell leukemia virus type 1 associated inflammatory myopathies (HAIM) in Salvador, Brazil

**DOI:** 10.1371/journal.pntd.0013409

**Published:** 2025-08-20

**Authors:** Adriele Ribeiro França Viriato, Marcela Câmara Machado Costa, Edmar Zanoteli, Luiz Antônio R. de Freitas, Thessika Hialla Almeida Araújo, Ney Boa-Sorte, Maria Fernanda Rios Grassi, Bernardo Galvão-Castro

**Affiliations:** 1 Centro Integrativo e Multidisciplinar de HTLV, Centro de Neurociências Escola Bahiana de Medicina e Saúde Pública, Salvador, Brazil; 2 Faculdade de Medicina, Universidade de São Paulo (USP), São Paulo, Brazil; 3 Faculdade de Medicina da Bahia, Universidade Federal da Bahia, Salvador, Brazil; 4 Instituto Gonçalo Moniz, Fundação Oswaldo Cruz, Salvador, Brazil; NIAID Integrated Research Facility, UNITED STATES OF AMERICA

## Abstract

**Background:**

Human T-cell leukemia virus type 1 (HTLV-1) causes inflammatory diseases and is associated with various muscle abnormalities, including polymyositis. Elevated serum creatine kinase (CPK) levels are typically indicative of muscle damage.

**Aims:**

This study aimed to determine the prevalence of HTLV-1 associated inflammatory myopathies (HAIM) in a large cohort of People living with HTLV-1 from Salvador, Brazil. Additionally, we sought to describe the clinical, laboratory, and histopathological findings of seven HTLV-1-infected patients with persistent hyperCKemia.

**Methods:**

This study included 503 HTLV-1-infected patients from a cohort in Salvador, Brazil, who were analyzed for creatine phosphokinase (CPK) levels. Clinical, laboratory, and pathologic examinations were performed in patients whose CPK levels were above the upper limit of the normal range in the two tests performed at different time points.

**Results:**

Polymyositis was the main cause of HAIM in the study population, with a prevalence rate of 0.6%. Two cases were diagnosed with muscular dystrophy and mitochondrial disease, and in two other patients the cause of hyperCKemia could not be determined and is currently under investigation.

**Conclusion:**

Polymyositis was the main cause of HAIM in this cfohort of People living with HTLV-1.

## Author summary

Human T-cell leukemia virus type 1 (HTLV-1) is a significant global health concern, affecting an estimated 10 million individuals worldwide, primarily in low- and middle-income countries. This virus can lead to severe diseases, including adult T-cell leukemia/lymphoma and a range of inflammatory conditions. One such condition is HTLV-1-associated myelopathy/spastic paraparesis and others include HTLV-1 associated inflammatory myopathies such as polymyositis, inclusion body myositis, and dermatomyositis. These diseases are characterized by skeletal muscle weakness, myalgia, and inflammation, resulting in functional impairment and increased morbidity.

The prevalence of HTLV-1-associated polymyositis varies widely across endemic regions. For instance, studies have shown a high prevalence in Jamaica (63–85%) but a lower prevalence in Japan (approximately 30%). In this study investigated the prevalence HAIM in a large cohort of people living with HTLV-1 in an endemic area of this infection in Brazil. We found that polymyositis was the main cause of HAIM. We recommend that patients infected with HTLV-1 who show clinical signs of myopathy should be promptly screened for HAIM.

## Introduction

The Human T-cell leukemia virus type 1 (HTLV-1) affects a significant number of people, with an estimated 10 million individuals infected worldwide, predominantly in low- and middle-income countries [1,2]. Brazil, particularly the state of Bahia in northeastern Brazil, is considered one of the regions with the highest prevalence of HTLV-1, contributing to Brazil having the highest absolute number of HTLV-1 cases globally [1,3]. Salvador, the capital of Bahia, is particularly affected with approximately 2% of the population living with HTLV-1 [4,5]. HTLV-1 causes severe diseases, such as adult T-cell leukemia/lymphoma (ATLL), a proliferative disease with high mortality, and a number of other inflammatory diseases, of which HTLV-1-associated myelopathy/spastic paraparesis (HAM/TSP) is the best known [6]. HAM/TSP is a chronic, progressive demyelinating disease characterized by spastic paraparesis, muscle weakness, and lower limb stiffness, affecting approximately 2–5% of People living with HTLV-1 [7]. HTLV-1-associated inflammatory myopathies (HAIM) have also been observed, including polymyositis [6,8,9], inclusion body myositis (IBM) [10–13], and, less commonly, dermatomyositis (DM) [14]. HAIM is characterized by skeletal muscle weakness, myalgia, and inflammation, which leads to functional impairment and increased morbidity. Elevated creatine phosphokinase (CPK) levels are frequently observed under these conditions. CPK, an enzyme critical for muscle function, serves as an indicator of muscle disease when elevated (hyperCKemia) [15]. Therefore, the evaluation of hyperCKemia along with other clinical features is crucial for diagnosing muscle disease. The most prevalent form of HAIM is polymyositis, frequently identified by hyperCKemia, muscle weakness primarily affecting the pelvic and scapular regions, and myalgia [6,8,9]. Polymyositis has been associated with HTLV-1 infection, particularly in regions considered endemic to this virus. For example, in Jamaica, 63%-85% of patients clinically diagnosed with polymyositis are infected with HTLV-1, and in Japan, the figure is approximately 30% [8,16,17]. In Brazil, few reports have described muscular involvement, mainly polymyositis, in HTLV-1 infected patients [18]. This study aimed to determine the prevalence of HAIM in a large cohort of People living with HTLV-1 in Salvador, Brazil. In addition, a case series of seven HTLV-1-infected patients with persistent hypercalcemia was described.

## Methods

### Ethics statement

The study was approved by the Institutional Research Board of the Escola Bahiana de Medicina e Saúde Pública (CAAE: 70078517.6.0000.5544), and informed written consent was obtained from participants when possible. In accordance with the ethical principles outlined in Brazilian regulations, the need for consent was waived only in cases of death or loss to follow-up.

The participants in this study were People living with HTLV-1 treated at the Integrative and Multidisciplinary Center for HTLV (CHTLV) between 2016 and 2018. The CHTLV outpatient clinic provides comprehensive biopsychosocial care to the public and is supported by public health services of the Brazilian National Health System (Sistema Único de Saúde [SUS]) [19]. HTLV-1 infection was confirmed by Elisa and Western blotting. For the determination of CPK levels, the Siemens Atellica CH Creatine Kinase (CK_L) assay was employed, utilizing upper reference limits of 192.0 U/L for females and 308.0 U/L for males.

The inclusion criteria were elevated creatine phosphokinase (CPK) levels above the upper limit on two separate tests at different time points. Participants who had taken myotoxic medications or were co-infected with HIV, hepatitis B, hepatitis C, syphilis, or toxoplasmosis were excluded from this study. A neurological examination was performed by a single neurologist (ARFV) for all included patients, and the diagnosis of HAM/TSP was based on Castro Costa’s criteria [20]. A comprehensive series of laboratory tests was performed as part of the study. These tests included the measurement of several parameters, including CPK, which is important in the assessment of muscle damage; lactate dehydrogenase (LDH), an enzyme that is elevated in several diseases, including muscle disease; alanine aminotransferase (ALT) and aspartate aminotransferase (AST), enzymes that indicate liver cell damage; and serologies for various infectious diseases, such as anti-HIV, anti-hepatitis B, anti-hepatitis C, syphilis, cytomegalovirus, and toxoplasmosis; antinuclear factor (FAN), an antibody that may be present in autoimmune diseases; antihistidy l antibody (anti-Jo1), an antibody associated with certain autoimmune muscle diseases and vitamin B12 levels: to exclude myelopathy due to B12 deficiency. Measurement of HTLV-1 proviral load (PVL), which determines the amount of HTLV-1 genetic material integrated into the genome of peripheral blood mononuclear cells (PBMCs). DNA was extracted using a Spin Column DNA Extraction System (Qiagen, Hilden, Germany). PVL was quantified in real time using the TaqMan polymerase chain reaction (PCR), as previously described [21]. Muscle biopsies of the left deltoid muscle were performed using an open technique. The samples were analyzed using the following techniques: 1) conventional histology of sections stained with Hematoxylin & Eosin, periodic acid-Schiff for glycogen, and modified Gomori trichrome for connective tissue matrix; 2) histochemistry with oil red 0 for fat and techniques for evaluating the enzymatic activity of succinate dehydrogenase, nicotinamide adenine dinucleotide, ATPase at pH 9.4, 4.3, and 4.6, alkaline phosphatase, acid phosphatase, and cytochrome C oxidase (COX); 3) immunohistochemistry using the indirect peroxidase method with primary antibodies against dystrophins (D1, D2, D3), merosin, dysferlin, CD4/CD8 lymphocytes, CD68 macrophages, and major histocompatibility complex class I (MHCI). The following parameters were used to standardize the final muscle biopsy report: variation in muscle fiber size, perimysial and endomysial fibrosis, number of nuclei and their position in the muscle cells (peripheral or central), presence of necrosis, presence of inflammatory cell infiltration, assessment of the internal architecture of the muscle cells, preservation of the mosaic pattern related to the distribution of the different types of muscle fibers, accumulation of fat and glycogen, acid phosphatase activity, accumulation of fat and glycogen, preservation of mosaic pattern by distribution of fiber types, and presence of COX-negative fibers. When indicated, a molecular panel for neuromuscular diseases [22] was used to examine the muscular dystrophies. Polymyositis was diagnosed according to the diagnostic criteria of the European Alliance of Associations for Rheumatology (EULAR) and the American College of Rheumatology (ACR) [23]; These criteria include various clinical, laboratory, and histopathological aspects for the evaluation and diagnosis of muscle diseases. Clinical aspects include the pattern of muscle weakness, age at symptom onset, and involvement of the cervical flexors. Laboratory aspects included evaluation of hyperCKemia, elevated transaminase levels, and the presence of positive anti-Jo1 antibodies. Histopathological findings included inflammatory infiltrates of mononuclear cells in the endomysium.

## Results

**Fig 1:**
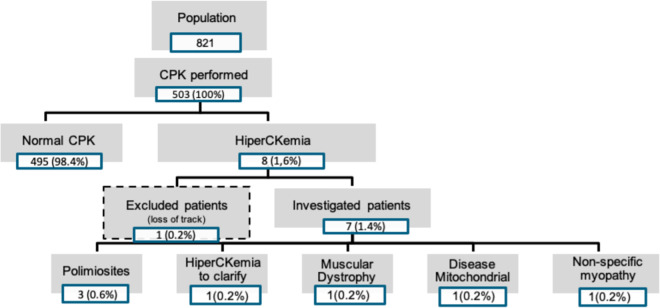
Flowchart of the evaluation of HiperCKemia in HTLV-1 infected patients.

Of the 821 patients examined during the study period, 503 underwent serological CPK testing (Fig 1). HyperCKemia was detected in 8 patients (0.62%). Seven of these patients underwent neurological consultation, resulting in the following diagnoses: polymyositis (three cases, 0.6%), muscular dystrophy (one case), mitochondrial disease (one case), nonspecific myopathy (one case), and hyperCKemia of unknown etiology (one case) (Fig 1). Five patients had definite HAM/TSP, and two patients showed no signs or symptoms of myelopathy (Table 1).

**Table 1:** Age, age of onset of symptom, sex and clinical features of patients.

Patient	Sex	Skin color	Age of onset symptoms (years)	Age at biopsy (years)	Length of disease (years)	Localization of symptoms^a^	CK (IU/L)	Diagnosis	Other HTLV-1 diseases^b^	Anti Jo1	Proviral load 10^6^/PBMC
1	F	Black	43	54	11	Prox. m. (UL,LL) NEM	655	PM	HAM/TSP	N	16.178x10^6^
2	F	Brown	41	57	16	Prox. m. (UL,LL)	610	PM	None	N	5.957x10^6^
3	M	Brown	59	ND	4	Prox. m. (UL,LL) NEM	1125	PM	HAM/TSP	N	ND
4	F	White	30	40	10	Prox. m. (UL,LL) NEM	828	Muscular Dystrophy	HAM/TSP	N	31.888x10 ^6^
5	F	Brown	29	35	6	Prox. m. (UL,LL)	1255	IM	HAM/TSP	N	10.959x10^6^
6	M	Black	49	73	24	Prox. m. (UL,LL)	1337	MD	HAM/TSP	N	111.312x10^6^
7	F	Brown	ND	ND	ND	None	9648	IH	None	N	80.308x10^6^

CK: plasmatic serum creatine kinase level; HTLV -1: Human T- lymphotropic virus, type 1; Anti Jo 1: anti histidil- tRNA sintetase; PM: polymyositis; IM: non- specific myopathy; MD: mitochondrial disease; IH: idiopathic hyperCKemia. Skin color self-referred.

^a^Weakness were generally symmetrical and localized in proximal muscles (Prox. m.), from the upper limbs (UL), lower limbs (LL) or both (UL, LL).

^b^HAM/TSP: HTLV-1 associated myelopathy/ tropical spastic paraparesis.

ND- Not Done.

### Description of the cases

**Fig 2:**
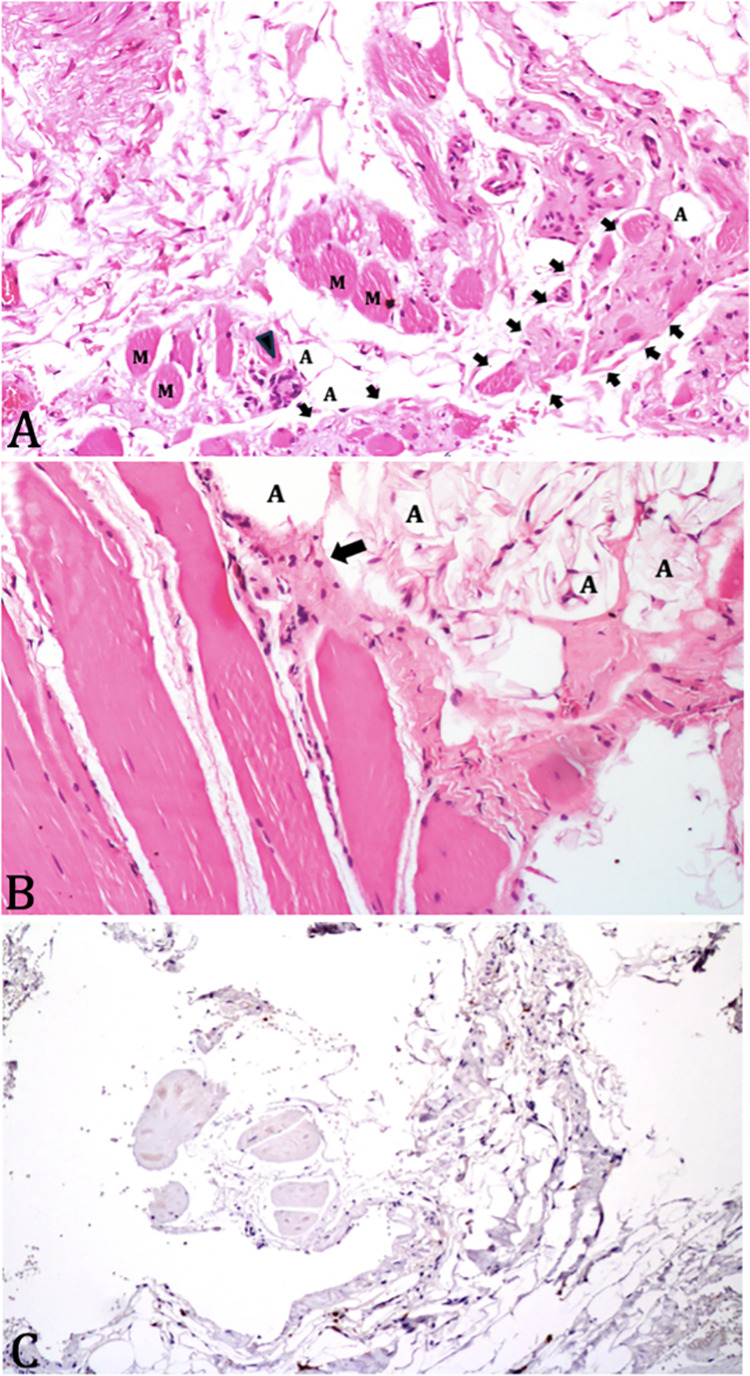
A) Case 1: The arrows delimit an area in which muscle fibers (M) are separated by fibrosis. The muscle fibers have varying sizes and shapes. There is a focus of inflammatory infiltration of mononuclear cells (arrowhead) and there are adipocytes (A) permeating the muscle (Hematoxylin-eosin staining); B) Case 2: A focus of inflammatory infiltration of mononuclear cells can be observed in the endomysium (arrow) and adipocytes (A) are seen permeating the muscle. C) Case 2: Immunostaining shows CD8-positive lymphocytes permeating the endomysium.

**Case 1** concerns a 54-year-old Black woman who tested positive for HTLV-1 during a blood donation at the age of 40. At the age of 43 years, she began to experience fatigue and proximal muscle weakness, initially in the lower limbs, which then gradually progressed to the upper limbs. At this point, the patient was diagnosed with neurogenic bladder. Laboratory examination revealed a CPK level of 655 U/L. At the age of 45, she underwent a muscle biopsy, which revealed inflammatory myopathy. Thereafter, the patient received immunosuppressive treatment with methotrexate. At 52 years of age, the patient presented with tetraparesis, mainly affecting the proximal and upper limbs, along with weakness of the neck muscles. The symptoms worsened over time. In 2018, at 54 years, a new muscle biopsy was performed, which showed muscle degeneration, variations in fiber size and centralization of the nuclei, endomysial and perimysial fibrosis, and foci of inflammatory infiltrate in the endomysium (Fig 2A). Necrosis was not seen. Immunohistochemical analysis of the muscle tissue showed normal expression of proteins, such as dysferlin, dystrophin, merosin, sarcoglycans, collagen 6, and caveolin-3**. C**PK measured in the same month as the second muscle biopsy was 255 U/L.. HTLV PVL was 16,178 x 10^6^ PBMC, approximately two years after the second muscle biopsy Based on the clinical presentation and findings from muscle biopsies, the patient fulfilled the EULAR/ACR criteria for the diagnosis of polymyositis.

**Case 2** concerns a 56-year-old Brown woman who, at the age of 41, began to experience pain, fatigue and weakness in her lower limbs. At the age of 43, she was diagnosed with HTLV-1 infection. At the age of 46, her CPK level was elevated (610 U/L), and polymyositis was suspected. As a result, immunosuppressive treatment with methotrexate was prescribed. At the age of 55 years, the patient presented with grade III muscle strength in the proximal muscles of the lower and upper limbs, moderate weakness of the neck muscles and abolished deep reflexes. At this point, the HTLV-1 PVL was 5,957 x 10^6^ PBMC, and CPK was 122 U/L. A muscle biopsy showed a myopathic pattern with moderate variability in fiber size, an increase in endomysial and perimysial connective tissue, heightened nuclear centralization (Fig 2B), and increased labeling for CD8 + cells (Fig 2C) and MHC I in the lymphocytic perimysial inflammatory infiltrate. Based on clinical presentation and muscle biopsy findings, this patient also met the EULAR/ACR criteria for the diagnosis of polymyositis.

**Case 3** concerns a Brown male patient born in 1954 who was diagnosed with HTLV-1 infection at the age of 50 when he presented with a neurogenic bladder. At the age of 59, he began to experience proximal lower limb muscle weakness and recurrent falls. Due to elevated CPK levels (1,125 U/L), a diagnosis of myopathy was made, and the patient was subsequently treated with corticosteroids. At the age of 62 years, the patient presented with tetraparesis mainly affecting the proximal muscles of the lower limbs, axial weakness centered on the neck flexors, increased deep reflexes in all limbs, atrophy of the deltoid and supraspinatus muscles, and moderate spasticity in the lower limbs. The patient’s clinical condition deteriorated considerably and also affected the respiratory muscles. Non-invasive ventilatory support was initiated, but the patient eventually developed respiratory failure and died four months after the onset of respiratory symptoms. Although a muscle biopsy could not be performed, this patient met the EULAR/ACR criteria for the diagnosis of polymyositis due to the clinical presentation, elevated CPK levels and progressive deterioration of muscle strength. The development of respiratory muscle involvement emphasizes the severity of the disease. Despite treatment with corticosteroids, the patient died, likely due to complications related to respiratory failure. No HTLV-1 PVL testing was performed for this patient.

**Case 4** involves a White woman born in 1978 who developed proximal weakness, mainly in the lower limbs, at age 30. At age 34, she was serologically diagnosed with HTLV-1 infection associated with the appearance of a neurogenic bladder. In September 2016, at the age of 38, the patient was wheelchair-dependent, exhibiting Grade 3 muscle strength in the thighs, areflexia of the lower extremities, and an elevated CPK level of 828 U/L. At age 39, she presented with tetraparesis, marked weakness of the pelvic girdle and lower limbs, and normal deep reflexes in all limbs. As a result, she was confined to a wheelchair. Laboratory testing revealed a high HTLV-1 PVL of 31,888 x 10^6^ PBMC. A muscle biopsy showed a dystrophic appearance with significant variability in fiber size, increased connective tissue in the endomysium and perimysium, heightened nuclear centralization, rare necrotic fibers, cytoplasmic vacuolization, a moderate endomysial inflammatory infiltrate containing CD8^+^ and MHC I+ lymphocytes, as well CD68^+^ macrophages, and intermyofibrillar disorganization. Based on these findings, the diagnosis in this case was muscular dystrophy. The presence of dystrophic changes in the muscle cell along with the clinical presentation of progressive proximal weakness and the histopathologic features support the conclusion of muscular dystrophy as the underlying disease in this patient.

**Fig 3:**
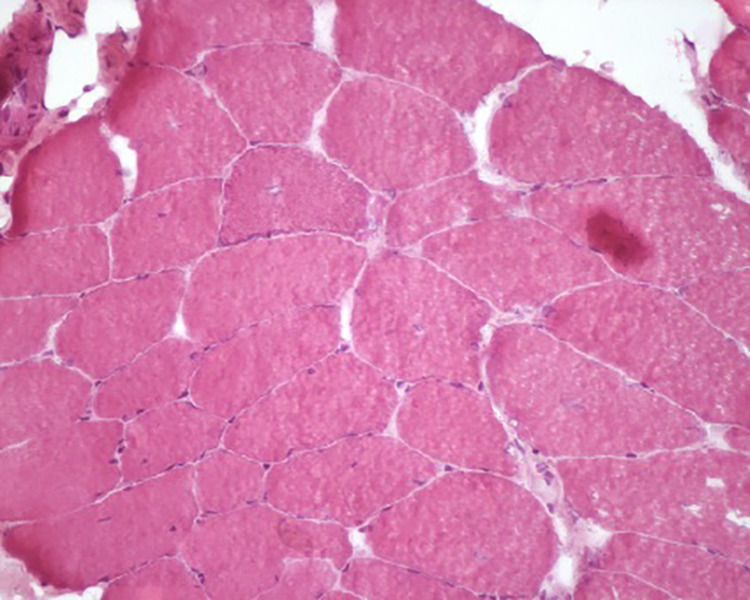
Case 5 - Histological section stained with Hematoxylin & Eosin shows muscle fiber with size variation.

**Case 5** concerns a Brown woman born in 1983 who was diagnosed with HTLV-1 infection during prenatal screening at the age of 27. At the age of 29, she began to present with diffuse muscle weakness accompanied by elevated CPK levels (1,255 U/L). At the age of 30, she was diagnosed with a neurogenic bladder. At the age of 35, she developed tetraparesis with more pronounced proximal weakness, spasticity in the lower limbs, markedly increased deep tendon reflexes in all four limbs, and bilateral positive Hoffman signs. The HTLV-1 PVL was 10,959 x 10^6^ PBMC. A muscle biopsy showed nonspecific myopathic changes characterized by variations in muscle fiber size and a tendency towards central core degeneration (Fig 3**).** Immunohistochemical labeling for dysferlin, dystrophins (D1, D2, D3), merosin, alpha-sarcoglycan, gamma-sarcoglycan, beta-sarcoglycan, collagen VI, caveolin-3 and desmin showed normal expression. Despite thorough examination, no specific myopathy was detected. At the time of the biopsy (October 2018), the patient was under methotrexate and prednisone therapy (40 mg/day). Electromyography was performed in February 2023 with normal results. CPK levels at age 40 were 1,059 U/L (March 2023) and 1,372 U/L (July 2023), and 862 U/L in April 2024 at age 41. No additional viral load testing was performed.

**Case 6** concerns a Black man born in 1945 who developed weakness in his lower limbs at the age of 49, which gradually spread to his upper limbs. At the age of 59, he tested positive for HTLV-1 during an examination for neurogenic bladder symptoms. An elevated CPK level (1337 U/L) was detected, leading to the suspicion of myopathy, and immunosuppressive therapy was initiated. Over time, his clinical condition deteriorated, accompanied by weight loss. At the age of 73, he was tetraparetic and confined to a wheelchair. The proximal lower limbs were primarily affected, and spasticity and increased reflexes were observed in all four limbs. He also had bilateral ptosis with restricted eye movement. The HTLV-1 PVL was elevated (111,312x10^6^ PBMC), however no prior PVL values were available, and CPK level was 137 U/L. A muscle biopsy revealed abnormalities in muscle fiber size, increased nuclear centralization with changes in the internal architecture of the fibers, and the absence of COX-positive fibers. Based on these findings, a diagnosis of mitochondrial disease was made. Nonetheless, the clinical features of spasticity and hyperreflexia, along with a high HTLV-1 PVL, raise the possibility that HTLV-1 infection contributed to progressive neuromuscular impairment, potentially exacerbating an underlying genetic myopathy.

**Case 7** concerns a Brown woman born in 1994 who was diagnosed with HTLV-1 infection at the age of 24 during a routine laboratory examination. Further investigations revealed hyperCKemia (CPK values of 9,648 U/I) and slightly elevated ALT and AST levels. No CPK isoforms were available. The HTLV-1 PVL was 80,308 x 10^6^ PBMC. Neurological examination was normal, with preserved muscle strength, reflexes, and sensitivity. There were no sphincter complaints. The muscle biopsy showed no abnormalities in the staining of dysferlin, dystrophins (D1, D2, D3), merosin, alpha-sarcoglycans, gamma-sarcoglycans, beta-sarcoglycans, collagen 6, caveolin-3 or desmin. No specific cause for her hyperCKemia was identified throughout the study.

In summary, histological examination of muscle tissue from the two cases of polymyositis (cases 1 and 2) revealed inflammatory myopathies. In case 1, the muscles exhibited advanced stages of degeneration, characterized by abnormal fiber size, increased endomysial and perimysial connective tissue, and foci of endomysial inflammatory infiltrates. Case 2 displayed variability in fiber size, increased endomysial and perimisial connective tissue, nuclear centralization, infiltration of CD68 + macrophages and MHC I expression by lymphocytes in the perimysial inflammatory infiltrate. One patient (case 4) was diagnosed with myofibrillar dystrophy. Muscle biopsy revealed variable fiber size, a tendency toward nuclear centralization, endomysial inflammatory infiltrate, and changes in the internal architecture of muscle fibers due to intermyofibrillar disorganization. Another patient (case 5) had nonspecific myopathy with mild variability in fiber size and an initial “moth-eaten” pattern in muscle tissue. Case 6 was diagnosed with mitochondrial myopathy characterized by variability in muscle fiber size, centralization of the nucleus, and mitochondrial alterations and the absence of COX-positive fibers. Cases 1, 2, and 3 were diagnosed as polymyositis, and cases 1, 3, 5, and 7 showed signs of myelopathy (Table 1).

## Discussion

To our knowledge, this is the first study to examine the prevalence of HAIM in a large cohort of People living with HTLV-1 with hyperCKemia. This finding is noteworthy as all previous research on the association between HTLV-1 and HAIM has been conducted in populations previously diagnosed with inflammatory myopathies. The results of this study show that polymyositis is the main cause of HAIM in this cohort (cases 1, 2 and 3). The detection of three cases of polymyositis in a sample of 503 patients, corresponding to 6,000 cases per million People living with HTLV-1, is strikingly high compared to the estimated incidence in the general population of about 5 cases per 1,000,000 [24].

The remaining cases were muscular dystrophy (case 4), non-specific myopathy (case 5), mitochondrial disease (case 6), and one case of hyperCKemia requiring further investigation (case 7). It is unlikely that muscular dystrophy and mitochondrial disease are related to HTLV-1 as they are mainly genetic. In the cases of non-specific myopathy and hyperCKemia (case 5 and 7), no definitive etiologic diagnosis could be made. Furthermore, no cases of HTLV-1-associated inclusion body myositis or HTLV-1-associated dermatomyositis were identified. Given the limited sample size of patients with inflammatory myopathies, it was more likely that polymyositis was identified, as this appears to be the most reported inflammatory myopathy in HTLV-infected individuals [9].

It should be emphasized that the diagnosis of polymyositis in this study was made according to the EULAR/ACR classification criteria, which ensure a stricter exclusion of differential diagnoses. Although the three polymyositis cases identified relied primarily on clinical features for diagnosis (with negative anti-Jo1 antibodies), muscle biopsies performed at later stages of disease and under immunosuppressive treatment nevertheless showed findings consistent with inflammatory polymyositis.

Only one patient (case 2) had clinical findings suggestive of an exclusive myopathy characterized by an anserine gait, proximal muscle weakness, and absence of pyramidal signs. The clinical features of the patients, including the fact that they were predominantly women, that myelopathy was present in two out of three cases, and that symptoms appeared after the age of 40 years, are consistent with the clinical-epidemiologic profile described in previous studies [16,25–29]. In addition, the two polymyositis cases showed advanced degenerative tissue changes, minimal inflammatory infiltration, and low HTLV-1 PVL levels, which is consistent with previous reports [9]. It is worth noting that one patient (case 3) developed respiratory failure and died four months after the onset of respiratory symptoms. Respiratory failure has been previously described as a complication of HTLV-I-associated myopathy [11].

The mechanism by which HTLV-1 can cause muscle damage is not yet fully understood. It is possible that HTLV-1, although it does not cause direct infection of the muscle fiber, triggers an immunopathological process mediated by T cells [30]. While rare previous studies have failed to detect viral particles in muscle fibers, the low HTLV-1 PVL and the lack of viral detection in muscle fibers support the idea that the process of muscle damage in polymyositis is immune-mediated and not directly caused by the virus itself [17]. In the present study, it was not possible to examine muscle samples to detect viral particles [31]. Although HTLV-1 likely did not contribute directly to the clinical progression in case 4, a potential role for HTLV-1 in triggering or exacerbating myopathy, as suggested by the dystrophic changes observed on muscle biopsy, cannot be entirely ruled out. Similarly, in cases without a definitive diagnosis of polymyositis (cases 5 and 7), it remains possible that HTLV-1 may have contributed to muscle damage.

Moreover, chronic activation of T cells induced by HTLV-1 may lead to the production of proinflammatory cytokines—such as IFN-γ, TNF-α, and IL-1β—which can contribute not only to skeletal muscle fiber injury but also, in some instances, to damage in cardiac muscle, brain tissue, and other muscle groups, potentially resulting in elevated CPK levels. However, in the present study, we found no clinical or laboratory evidence of involvement of other organs, such as the brain or heart.

It is also important to consider that elevated CPK levels may result from non-viral causes. Certain medications, particularly statins, are well-documented to induce myopathy and elevate CPK levels in susceptible individuals. Notably, none of the cases examined in this study reported prior statin use. Consistent with previous literature [8,9], the patients with polymyositis in this study showed a significant delay between symptom onset and diagnosis. Several factors may contribute to this delay, such as patients’ limited access to medical care, insufficient awareness of HAIM among medical professionals, and difficulties in performing muscle biopsies, particularly in low-resource settings.

One limitation of this study is the lack of longitudinal laboratory data, particularly with respect to CPK levels and HTLV-1 proviral load. As this was a retrospective study, serial measurements were not available, which limits our ability to assess trends over time or establish temporal associations between biochemical markers and clinical progression. Although PVL is generally stable in our clinical experience [32], a single CPK measurement may not accurately reflect a patient’s baseline muscle enzyme activity or the dynamics of muscle involvement. CPK measurement has been shown to be a valuable screening tool to detect potential muscle disease in People living with HTLV-1. Consequently, patients infected with HTLV-1 who show clinical signs of myopathy should be promptly screened for HAIM.

In conclusion, the measurement of CPK may serve as a valuable screening tool for the early detection of potential muscle disease in individuals infected with HTLV-1. We therefore recommend that patients with HTLV-1 infection who exhibit clinical signs of myopathy be promptly evaluated for HAIM. Furthermore, additional research involving diverse cohorts worldwide is essential to accurately determine the global prevalence of HAIM.
